# A conception of genetic parenthood

**DOI:** 10.1111/bioe.12493

**Published:** 2018-07-23

**Authors:** Thomas Douglas, Katrien Devolder

**Affiliations:** ^1^ Oxford Uehiro Centre for Practical Ethics Oxford United Kingdom

**Keywords:** assisted reproduction, cloning, genetic parenthood, Heidi Mertes, in vitro reproduction, mitochondrial replacement therapy, stem cell derived gametes

## Abstract

We seek to develop a plausible conception of genetic parenthood, taking a recent discussion by Heidi Mertes as our point of departure. Mertes considers two conceptions of genetic parenthood—one invoking genetic resemblance and the other genetic inheritance—and presents counter‐examples to both conceptions. We revise Mertes’ second conception so as to avoid these and related counter‐examples.

## 1 INTRODUCTION

Recent scientific developments suggest that it will become increasingly common for children to inherit their genomes from one or more existing human beings, but in ways that differ substantially from standard forms of human reproduction. One such development is the successful application of mitochondrial replacement therapy (MRT) in human reproduction. The aim of MRT is to avoid transmission of mitochondrial DNA (mtDNA) mutations; a baby is created through in vitro fertilization (IVF) using a man’s sperm and a woman’s egg, but shortly before or after fertilization, the egg’s disease‐linked mtDNA is replaced with healthy mtDNA from a donor egg. The first baby resulting from MRT was born in 2016. Such babies have been referred to as “three‐parent babies.”^1^

Another technique that may enable a significant departure from “normal” human genetic inheritance is the creation of stem cell‐derived gametes. In the not too distant future, sperm and eggs derived in the laboratory from pluripotent stem cells may be used to create embryos and eventually children. The pluripotent stem cells needed to generate these gametes could be extracted from embryos left over following fertility treatments, from embryos created through IVF for the specific purpose of gamete‐derivation, or from embryos cloned from a person’s somatic (body) cells, either via a process known as somatic cell nuclear transfer (SCNT^2^) or via the induced pluripotent stem (iPS) cell technique.^3^

Questions arise as to whether the children created through these techniques would have genetic parents, and, if so, who, precisely, those genetic parents would be. Bioethicists have already begun to address these questions,^4^ motivated in part by the thought that, if these children would be “genetic orphans” or would have three or more genetic parents, this could be ethically significant. For example, it has been suggested that creating genetic orphans might be problematic because genetic parenthood is sometimes regarded as a “last ditch” criterion for assigning the responsibilities of social parenthood,^5^ and that creating “three‐parent children” might be problematic because it is not clear whether the “third parent”—the donor of the mtDNA—would possess any of the rights or obligations of social parenthood.^6^

Despite this growing interest, however, there is little agreement on the nature of genetic parenthood, and indeed, few conceptions of genetic parenthood have been proposed. We believe a better understanding of genetic parenthood is needed for two reasons. First, *if *the genetic parenthood relation is morally significant, as some have suggested, then it is obviously important that we are able to determine when the relation is present and when it is not. Second, a fuller understanding of genetic parenthood may help to inform discussions of whether genetic parenthood is indeed morally significant. For example, if, on the correct understanding of genetic parenthood, the presence or absence of this relation depends on factors that are clearly morally irrelevant, this will cast serious doubt on the suggestion that genetic parenthood is morally significant.

In this article, we seek to develop a plausible conception of genetic parenthood. We take as our point of departure a recent discussion by Heidi Mertes,^7^ and draw also on some recent work by Robert Sparrow, who endorses many of Mertes’ views.^8^ In sections 2 and 3, we outline and assess two conceptions of genetic parenthood that are introduced and critiqued by Mertes. In section 4, we propose our own conception.

However, before turning to these tasks, we must offer three preliminary comments.

The first concerns the relationship of the concept of genetic parenthood to other concepts of parenthood. The concept of genetic parenthood that we seek to capture is, of course, distinct from the concepts of gestational and social parenthood. But we believe that it is also, though less obviously, distinct from the concepts of biological and causal parenthood. Biological and causal parenthood are, we take it, concepts that were without error deployed even before the details of genetic inheritance were understood.^9^ By contrast, the concept of genetic parenthood that we seek to capture is, we think, one that has developed in response to the acquisition of such understanding, and that now co‐exists with, though has arguably largely replaced in everyday discourse, biological and causal parenthood.

The second preliminary comment concerns the scope of our analysis. We assume throughout that genetic parenthood is a relation that obtains between two *humans*, and we discuss only cases involving humans. There is plausibly a concept of genetic parenthood that applies to other species; perhaps it applies to all other species that reproduce sexually. But we do not wish to commit ourselves to the view that this is the same concept as the concept that applies to humans.

Finally, the third clarification concerns the aim of our analysis. There are various aims one might have in formulating a conception of genetic parenthood. First, one might aim to capture the concept of genetic parenthood that is implicit in common usage of the term. What are the necessary and sufficient conditions of genetic parenthood as that concept is employed in everyday discourse? That is, under what conditions do people tend (not) to ascribe genetic parenthood? Second, one might seek to capture what people care about when they, for instance, pursue forms of assisted reproduction in preference to adoption. What sort of genetic relation do prospective social parents want to have with their social children?^10^ Note that this may not map on to the concept of genetic parenthood implicit in everyday discourse; for instance, one might seek fertility treatment to have some weak genetic link with the child even though people generally only ascribe genetic parenthood when a stronger link is present. Third, one might seek to capture what (if anything) is actually morally significant in the genetic relationship that typically exists between social parents and their social children. What aspects of this genetic relationship, if any, are objectively valuable? What aspects, if any, play a role in generating the rights and obligations of social parenthood?

Our aim will be to develop a conception of the first kind; we aim to formulate a conception of genetic parenthood that captures the concept of genetic parenthood implicit in everyday usage.

## 2 INFORMATIONAL OVERLAP

As noted above, we take as our starting point Mertes’ instructive discussion of genetic parenthood. Mertes distinguishes two candidate conceptions of genetic parenthood. According to the first of these conceptions,

a child is my genetic child when it has 50% of my DNA or when it has 23 of my chromosomes. This 50% overlap of genetic material is, for example, what is looked into when performing a paternity or maternity test.^11^

What does it mean to say that C “has 50% of P’s DNA” or that there is a “50% overlap” between C and P’s DNA? The thought cannot be that 50% of C’s DNA is numerically identical to 50% of P’s DNA, for P and C’s DNA are in different locations, and have different survival conditions (one could destroy P’s DNA without destroying C’s). Nor does Mertes mean that 50% of C’s DNA is *inherited from *P, for, as we shall see, she criticizes this account of genetic parenthood on the basis that it is insensitive to the direction of inheritance. Rather, the thought simply seems to be that 50% of C’s DNA resembles 50% of P’s DNA. More precisely the thought appears to be:

*Informational Overlap*: P is C’s genetic parent if and only if 50% of C’s genetic material *encodes the same information as *50% of P’s DNA.

One difficulty with this conception is that it is too inclusive. For instance, as Mertes notes, it would arguably classify one’s sibling as one’s parent (or child), or one’s parent as one’s child: “My siblings also share 50% of my DNA, as do my parents, and yet, the relation I have with my parents and siblings differs profoundly from the one I have with my children.”^12^ Similarly, as Sparrow has pointed out, this conception of genetic parenthood could imply the existence of a genetic parenthood relation between individuals who, though not close relatives, through chance have a 50% genetic overlap.^13^

A second difficulty is generated by the fact that “almost all (99.9%) of nucleotide bases are exactly the same in all humans.” This leads Mertes to question “whether it even makes sense to say that we share 50% of DNA with our genetic children.”^14^

We take issue with Mertes’ second concern. It makes perfectly good *sense* to say that we “share 50% of our DNA with our children,” in the sense specified by *Informational overlap*, it is just that this is false: we in fact share *much more* of our DNA with them than that, since we share more than 99% of our DNA even with other humans to whom we are not closely related. Moreover, it seems possible to modify *Informational overlap *to avoid this problem; we simply need to increase the percentage of informational overlap that is required. After all, whatever share of our DNA overlaps with that of all other humans, an even higher share overlaps with that of our putative genetic parents. Suppose that 99.9% of our DNA overlaps with that of all other humans, but 99.95% overlaps with that of our putative genetic parents. In that case, we should modify *Informational overlap *such that “50%” is replaced by “99.95%.”

Mertes’ first concern, however, would remain. *Informational overlap *does not distinguish parent–child relations from child–parent relations, some sibling–sibling relations, and instances in which genetic strangers by chance share the same proportion of genetic information as putative genetic parents and their putative genetic children. This is clearly a problem.

Moreover, this problem cannot be resolved by being more precise about *which *information in an individual’s genome must resemble that of the putative genetic parent. The nuclear genome of most humans is composed of 23 pairs of chromosomes, where in most cases one member of each and every chromosome pair resembles a corresponding chromosome possessed by the putative genetic parent. Perhaps we should revise *Informational Overlap *to require this specific form of genetic overlap. This might seem to help prevent siblings from falling within the scope of *Informational Overlap*, since any genetic overlap between siblings will not normally be distributed across chromosome pairs in the same way. Still, siblings could, by chance, possess the same sort of genetic overlap as that existing between putative genetic parents and their putative genetic children, as indeed could strangers. In addition, the proposed revision to *Informational Overlap *does not help to prevent the genetic parenthood relation from running in both directions, such that P is C’s genetic parent, and vice versa.

## 3 DIRECT GENETIC DESCENT

How should we solve these problems? One option that Mertes considers involves shifting to a second conception of genetic parenthood according to which “X is a genetic child of Y if X is directly derived from Y’s genes.”^15^

We take it that the “directly” is intended to rule out cases in which the genetic relation is one of genetic *grand*parenthood or some similarly or more distant genetic descent—that is, relations of genetic descent that are mediated by other individuals. (It would need to be further specified what sort of entities can qualify as “individuals” for the purposes of this directness condition, an issue to which we will return below.)

We take it also that the kind of derivation in question is one in which the putative genetic child’s *genes *derive from the putative genetic parent’s genes. Suppose a sperm from P1 is used to fertilize an egg from P2. The resulting zygote then has its DNA removed and replaced by DNA from some other individual T. This zygote is then carried to term and eventually a child, C, is born. There is a sense in which C derives directly from P1 and P2’s genes; those genes governed the development of gametes, which created a zygote from which C developed. But C’s *genes *do not derive from P1 and P2’s genes, they derive instead from T’s genes, and this surely prevents P1 and P2 from qualifying as C’s genetic parents.

Taking these two qualifications into account, we could formulate Mertes’ second proposed conception of genetic parenthood as follows:

*Direct Genetic Descent*: P is C’s genetic parent if and only if (a) C’s genes derived from P’s genes, and (b) not through deriving from the genes of some third, intervening individual, M.

What should we make of this conception? Mertes holds that it is susceptible to a number of counter‐examples. For instance, she asks us to imagine a case (call this *Cloned Child*) in which P1 and P2 have a child C1 whom they then clone to create C2. According to *Direct genetic descent,* C2 is C1’s genetic child, but Mertes thinks it seems more plausible to say that C2 is the genetic child of P1 and P2. Sparrow would likewise deny that C2 is the child of C1, for he holds that “clones share too much genetic material with their donors to be their genetic children.”^16^ He appears to concur with Mertes’ view that P1 and P2 are the genetic parents, writing that, “[i]n such cases, cloning clearly produces a child genetically related to its parents in the same way as a child that they might— indeed, did—conceive naturally… It produces ‘genetic offspring’.”^17^

In the second case (call this *Cloned Parent*), P1 and P2 decide to directly clone P1 instead, creating a child C3. *Direct Genetic Descent *implies that P1 is C3’s genetic parent, but Mertes questions whether this is so; she thinks C3 may bear *too much *genetic resemblance to P1, and again, Sparrow concurs, suggesting that the relation between P1 and C3 is closer to that between identical twins than to that between genetic parents and their genetic children.

Finally, in the third case (call this *Mitochondrial Donation*), a child (C4) derives all of her nuclear DNA in the usual way from P1 and P2, but her mitochondrial DNA, which constitutes about 0.15% of her total genome, comes from a third individual (P3). *Direct Genetic Descent *implies that P3 is C4’s genetic parent, but Mertes thinks that, in many circumstances, this is not intuitively plausible.^18^

We do not ourselves have clear intuitions about genetic parenthood in the *Cloned Child*, *Cloned Parent *and *Mitochondrial Donation *cases. But let us suppose that Mertes’ and Sparrow’s intuitions here are veridical. What would follow? Mertes’ own conclusion is that we should reject both *Informational Overlap *and *Direct Genetic Descent. *She holds that

Rather than being a black‐or‐white concept (either one is a genetic parent or one is not), it appears that there is also a grey area in which some people may be more or less of a genetic parent than others. There is no fixed, scientific, everlasting criterion of genetic parenthood that everyone can agree upon. Quite on the contrary, the concept is increasingly challenged by new and hypothetical interventions in reproductive medicine.^19^

This passage advances a number of claims. Among them, plausibly, are the claims that:

1. Genetic parenthood comes in degrees or at least has vague boundaries.
2. There is disagreement about the nature of genetic parenthood.
3. Genetic parenthood is a subjective concept that depends on the views of people about what sorts of genetic relation matter.
4. Those views may change as a result of scientific developments.

We believe that these claims are all somewhat plausible, but we do not think they follow from the rejection of *Informational Overlap *and *Direct Genetic Descent*. In particular, we think it would be premature to conclude from the rejection of these views that genetic parenthood is a subjective concept in the sense specified by 3 (and assuming that we are interested in the concept of genetic parenthood implicit in common usage), for there are other objective conceptions of genetic parenthood available. In the next section, we explore some of these available conceptions and present one of these as a plausible alternative to *Informational Overlap *and *Direct Genetic Descent*.

## 4 OUR CONCEPTION

One promising route to a more plausible conception of genetic parenthood would involve refining *Direct Genetic Descent *so as to avoid the putative counter‐examples that Mertes and Sparrow discuss. Here is one possible refinement:

*Direct Gametic Genetic Descent*: P is C’s genetic parent if and only if (a) C’s genes derived from P’s genes, (b) through a gamete produced by P, and (c) not through deriving from the genes of some third, intervening individual, M.

The requirement that genetic derivation occurs via a gamete prevents cloning (whether through SCNT or an iPS cell‐based technique) from generating a relation of genetic parenthood between the donor individual and the clone. Thus, it avoids the putatively implausible implications that C1 is the genetic parent of C2 in *Cloned Child *and P1 is the genetic parent of C3 in *Cloned Parents*. It also allows us to evade the conclusion that P3 (the mitochondrial donor) is the genetic parent of C4 (the mitochondrial recipient) in *Mitochondrial Donation.*

However, though *Direct Gametic Genetic Descent *may allow us to avoid implausible attributions of genetic parenthood in these cases, it arguably does entail implausible *denials *of genetic parenthood. It implies that the clones created in *Cloned Child *and *Cloned Parent *lack any genetic parents, since their genomes were not inherited via gametes. Yet, Mertes and Sparrow believe that these individuals do have genetic parents: on their view, P1 and P2, the genetic parents of the donor individual (C1), are also the genetic parents of the clone (C2) in *Cloned Child. *In *Cloned Parent, *the genetic parents of the donor (P1) are presumably again the genetic parents of the clone (C3). In denying that the clones created in these cases have genetic parents, *Direct Gametic Genetic Descent *may seem too stringent in the conditions that it imposes on genetic parenthood.

Moreover, it is possible to imagine circumstances closer to those of normal human reproduction that also create difficulties for *Direct Gametic Genetic Descent*—cases in which humans would reproduce without the creation of gametes, but in which we would still want to ascribe genetic parenthood. Suppose it were possible for adult humans to reproduce through a process that we might call *Two‐Donor Genome Transplantation. *In this process, DNA from a zygote would be replaced with genetic material extracted directly from body cells taken from two individuals, P1 and P2, who would each contribute roughly 50% of the zygote’s (new) genome. If the embryo derived from this zygote were carried to term, we would want to say that P1 and P2 were the genetic parents of the resulting child, even though no gametes were involved in the reproductive process. *Direct gametic genetic descent *cannot accommodate this verdict.

This second problem could be avoided by replacing the requirement for genetic inheritance *via gametes *with a requirement for genetic inheritance of some specified proportion of the putative genetic child’s genome from the putative genetic parent. Consider:

*Direct Proportionate Genetic Descent*: P is C’s genetic parent if and only if (a) *some proportion X* of C’s genes derived from P’s genes and (b) not through deriving from the genes of some third, intervening individual, M.^20^

Suppose we set “proportion X” to “20–80%.”^21^ In that case, like *Direct gametic genetic descent*, this formulation avoids the implication that donor individuals are the genetic parents of the clones created from them (the proportion of genes inherited by the clones is too high) as well as the implication that mitochondrial donors are the genetic parents of the mitochondrial recipients (the proportion of genes inherited by the recipients is too low). Yet, unlike *Direct Gametic Genetic Descent*, this formulation also gets the right result in *Two‐Donor Genome Transplantation*; it implies that the two genetic donors in this process are the genetic parents of the resulting child.

However, *Direct Proportionate Genetic Descent *remains too stringent in the cases of *Cloned Child *and *Cloned Parent*, for it continues to imply that the clones produced in these cases have no genetic parents—an implication that Mertes and Sparrow find implausible. On *Direct Proportionate Genetic Descent*, the donor does not count as the genetic parent of the clone because she passes on too much of her genetic material. But the genetic parents of the donor seemingly also do not count as the parents of the clone, because their genetic contribution is mediated by another individual: the donor. Thus, the directness requirement rules out their genetic parenthood of the clone.

To avoid this implication, it is necessary to weaken the directness requirement. There are various plausible ways in which it could be weakened; we shall make what we think is the most minimal plausible revision required to yield the result that Mertes and Sparrow prefer in *Cloned Child *and *Cloned Parent*. Our suggested refinement consists in qualifying how the intervening individual should genetically relate to his or her descendant in order to break or maintain a presumed genetic parenthood relation between the ancestor and the descendant. In *Cloned Child, *Mertes and Sparrow share the intuition that P1 and P2, not C1, qualify as the genetic parents of C2 (who is cloned from C1). We propose that the intervening individual (C1) does not break the genetic parenthood relation in this case because she passes on *too much *of her genetic information to C2. This appears to be the result that Mertes and Sparrow favour. In *Cloned Parent*, Mertes and Sparrow think it is counterintuitive that C3 (a clone of P1) has no genetic parents; they appear to believe that P1’s genetic parents count as C3’s genetic parents too. We propose that this is because P1 again passes on too much of her genetic information to C3 and so does not qualify as an intervening individual for the purposes of breaking the genetic parenthood relation. We stipulate, then, that, when C derives genetic information from P via some intervening individual M, this disqualifies P from being C’s genetic parent only if C derived some specified proportion of his genetic material from M. This specified proportion will most plausibly, as with “proportion X”, be a *range *of proportions, and it may be a range with fuzzy boundaries. But so long as the range is set so as to exclude cases of cloning—that is, cases where (virtually) 100% of C’s genes derive from M’s genes—we can maintain the result that, if C is a clone of M, the genetic parenthood relation between P and C is preserved.

This suggested revision yields the following conception of genetic parenthood:

*Modified Direct Proportionate Genetic Descent*: P is C’s genetic parent if and only if (a) *some proportion X* of C’s genes derived from P’s genes and (b) not through deriving from the genes of some third, intervening individual M *from whom C derived proportion Y of his genes.* 22

*Modified Direct Proportionate Genetic Descent* is our favoured conception of genetic parenthood. We do not claim that it is certainly correct; it may well be susceptible to counter‐examples that we have not identified, and in any case, it is not clear that there is any single concept of genetic parenthood implicit in everyday usage. But we do believe that *Modified Direct Proportionate Genetic Descent* is an *initially plausible *conception of genetic parenthood, and that it is more plausible than any of the alternative conceptions described above. We thus propose that it could serve as a helpful starting point for future analyses.^23^

Of course, this conception needs to be fleshed out in various ways. Most obviously, more would need to be said about what ranges of proportions are picked out by X and Y, and whether these ranges of proportions have sharp boundaries. Similarly, more needs to be said about what types of entities can fill the roles of P, M and C on our conception. For example, must these be live born humans, or can they be filled by, for example, human zygotes, embryos or foetuses? One plausible view would be that only human *organisms* can fill these roles, but this does not settle all of the interesting questions here, since there is, for example, disagreement about whether zygotes and early embryos qualify as human organisms.^24^

Then there is the question of moral significance. It is not our intention to explore in detail the moral implications of this conception of genetic parenthood here—our goal in this article has been conceptual, not ethical. However, it is perhaps worth briefly noting that our conception does not appear helpful to those who wish to hold that the genetic parenthood relation is of moral significance. The chief reason for this is that *Modified Direct Proportionate Genetic Descent *invokes properties that are most naturally understood as threshold (or “range”) properties: namely, the property of deriving proportion X (or Y) of one’s genes from another individual. These properties are most naturally understood to consist in lying between two (possibly fuzzy) thresholds on some *scale* of genetic inheritance. Yet, it is difficult to see why these thresholds should mark anything of moral significance. Perhaps it matters morally that we derived *some *proportion of our genes from another individual. Perhaps it even matters that we derived a greater rather than a lesser proportion from that other individual. But even if the proportion of our genetic inheritance matters in these ways—a point on which we remain neutral—it is difficult to see why it should matter that this proportion lies between two thresholds marking the boundaries of our commonsense conception of genetic parenthood. Those thresholds seem arbitrary from a moral point of view.^25^

## CONFLICT OF INTEREST

The author declares no conflict of interest.

## Footnotes

1 For example, Sample, I. (2015, February 2). “Three‐parent” babies explained: What are the concerns and are they justified? *The Guardian*. Retrieved February 9, 2018, from https://www.theguardian.com/science/2015/feb/02/three-parent-babies-explained.

2 SCNT involves transferring the nucleus of a somatic cell into an egg cell from which the nucleus and thus most of the DNA has been removed. The manipulated egg cell is then treated with an electric current in order to stimulate cell division, resulting in the formation of an embryo that is almost genetically identical to the somatic cell donor.

3 The induced pluripotent stem cell technique involves the direct reprogramming of somatic cells to an embryonic state through genetic manipulation. See, for example, Takahashi, K., Tanabe, K., Ohnuki, M., Narita, M., Ichisaka, T., Tomoda, K., & Yamanaka, S. (2007). Induction of pluripotent stem cells from adult human fibroblasts by defined factors. *Cell*, *131*(5), 861–872.

4 See, for example, Mertes H., & Pennings, G. (2008). Embryonic stem cell derived gametes and genetic parenthood: A problematic relationship. *Cambridge Quarterly of Healthcare Ethics*, *17*(1), 7–14; Bredenoord, A. L., Dondorp, W., Pennings, G., & De Wert, G. (2011). Ethics of modifying the mitochondrial genome. *Journal of Medical Ethics*, *37*, 97–100; Sparrow, R. (2012). Orphaned at conception: The uncanny offspring of embryos. *Bioethics*, *26*(4), 173–181; Mertes, H. (2014). Gamete derivation from stem cells: Revisiting the concept of genetic parenthood. *Journal of Medical Ethics*, *40*(11), 744–747; Palacios‐González, C. (2017). Does egg donation for mitochondrial replacement techniques generate parental responsibilities? *Journal of Medical Ethics*, published online first: 25 October 2017. doi:10.1136/medethics‐2017‐104400; Piotrowska, M. (2018) Is ‘assisted reproduction’ reproduction? *The Philosophical Quarterly*, *68*(270), 138–157.

5 Sparrow, *op. cit.* note 4, pp. 178–179.

6 Loike, J. D., & Reame, N. (2016, December 22). Opinion: Ethical considerations of ‘three‐parent’ babies. *The Scientist*. Retrieved February 8, 2018, from https://www.the-scientist.com/?articles.view/articleNo/47725/title/Opinion--Ethical-Considerations-of--Three-Parent--Babies/;Palacios-Gonzalez, *op. cit.* note 4.

7 Mertes, *op. cit.* note 4.

8 See, for example, Sparrow, *op. cit.* note 4, p. 177.

9 We thank an anonymous reviewer for this point regarding biological parenthood.

10 This seems to be at least in part Mertes’ aim in Mertes, *op. cit.* note 4.

11 Ibid., p. 744. See also Sparrow, R. (2006). Cloning, parenthood, and genetic relatedness. *Bioethics*, *20*(6), 308–318: 313–314.

12 Mertes, *op. cit.* note 4, p. 744. See also Sparrow, *op. cit*. note 11, who writes that, “the ‘genetic relation’ between siblings, who share roughly half their DNA with each other, is the same as the relation between parents and their children. Yet this relation is not one of parenthood.”

13 Sparrow, *op. cit.* note 11, p. 314, writes that, “[a] concern with genetic similarity will even allow total strangers to be ‘more related’ to us than our own children. While, statistically, my child is likely to have a phenotype (partially) determined by the expression of 50% of my genes, their character may in fact reflect the expression of many more, or less, of my genes. Moreover, while the particular set of genes that my cells contain – my total genetic make‐up – is almost certainly unique, the individual genes within that set are not. Except for the small number of mutations that occur with each conception, I share all my individual genes with thousands, and probably millions, of other people. Any of these people may in fact have more of “my” genes than my own children.” Alpern makes a similar point in Alpern, K. D. (1992). Genetic puzzles and stork stories. In K. D. Alpern (Ed.), *The ethics of reproductive technology* (pp. 147–169). Oxford, UK: Oxford University Press.

14 Mertes, *op. cit*. note 4. Relatedly, Sparrow, *op. cit*. note 11, p. 313, notes that “[g]iven that all human beings share the vast majority of their DNA with each other (as well as with rabbits, fish and bacterium), ‘half their genes’ here can only refer to the genetic variation within the human population.”

15 Mertes, *op. cit.* note 4. This definition is proposed by Avery Kolers in Kolers, A. (2003). Cloning and genetic parenthood. *Cambridge Quarterly of Healthcare Ethics*, *12*(4), 401–410.

16 Sparrow, *op. cit.* note 11, p. 313.

17 Sparrow, R. (2009). Therapeutic cloning and reproductive liberty. *The Journal of Medicine and Philosophy*, *34*(2), 102–118, p. 106.

18 Mertes, *op. cit.* note 4, p. 745. Mertes is more willing to assign genetic parenthood to P3 in cases where P3 and P1 are in a personal relationship with one another, will be C4’s social parents, and pursue mitochondrial donation in part to ensure that their child will have some genetic link to P3. In this case, P2 effectively serves only as a nuclear DNA donor. Mertes writes that, in this situation, despite the small genetic contribution made by P3 to C4, P3 might ‘consider herself’ a genetic parent of C4. (Sparrow also thinks that intentions sometimes matter for assigning genetic parenthood. See Sparrow, *op. cit.* note 11, pp. 314–316.) We agree that P3 might consider herself a genetic parent in this case, but we do not find it plausible that she *is *a genetic parent on the concept of genetic parenthood implied by common usage. We suspect that intuitions to the effect that P3 is a genetic parent in this variant of the case may be influenced by intuitions about *social *parenthood that are in fact irrelevant to genetic parenthood. For other views on mitochondrial donation and parenthood, see, for example, Baylis, F. (2013). The ethics of creating children with three genetic parents. *Reproductive BioMedicine Online*, *26*(6), 531–534; Cohen, J., & Alikani, M. The biological basis for defining bi‐parental or tri‐parental origin of offspring from cytoplasmic and spindle transfer. *Reproductive BioMedicine Online*, *26*(6), 535–537; Garasic, M. D., & Sperling, D. (2015). Mitochondrial replacement therapy and parenthood. *Global Bioethics*, *26*(3–4), 198–205.

19 Mertes, *op. cit.* note 4, p. 745.

20 Note that, although this formulation, like *Informational Overlap*, makes reference to a proportion, this proportion implies a causal relation, not a relation of resemblance; roughly 50% of the child’s genes must *derive from*, not necessarily resemble, the parent’s genome. Thus, suppose P1 and P2 want a child, but P2 is infertile. They create a child, C, using P1’s sperm and an oocyte from P2’s identical twin sister. Though roughly 50% of C’s genes will resemble P2’s genes, they were not derived from her genes. Thus, on *Direct Proportionate Genetic Descent*, P2 is not a genetic parent of C5, though her twin sister is.

21 As noted by Palacios‐González, *op. cit.* note 4, some genes may have a much more substantial impact on the child’s phenotype (e.g. skin colour) than others. One solution could be to weight the proportion of genes of the putative genetic parent for genetic importance, so that some genes count more than others.

22 Note that it might be thought that proportions X and Y should be identical. In that case, setting aside further intervening generations, M will break the genetic parenthood relation between P and C if and only if C is the genetic child of M.

23 In a recent paper, Piotrowska, *op. cit.* note 4, p. 155, argues that, “we have a genetic parent‐offspring relation when what is transmitted from one generation to the next involves material overlap, contributes to the phenotype of the offspring and persists to the future generation.” She understands material overlap such that it requires the transfer of physical matter. We reject the view that material overlap is an essential requirement for genetic parenthood. Suppose that, to contribute its genetic information to an egg cell, a sperm cell merely has to touch that egg. The egg is able to detect the genetic information of the sperm via some form of electromagnetic communication, and it then internally creates genetic material encoding that same information. Thus, the sperm passes on its genetic information to the egg, but without any transfer of physical matter. No molecules or particles from the sperm enter the egg. It seems to us that this mode of genetic heritance would be consistent with the presence of a genetic parenthood relation between the individual who produced the sperm and the individual whom the fertilized egg becomes.

24 For a denial of the view that early embryos qualify as human organisms, see Persson, I. (2003). Two claims about potential human beings. *Bioethics*, *17*(5‐6), 503–517.

25 We thank an anonymous reviewer for pressing us to make this point.

